# Recent Progress on Nanocarriers for Topical-Mediated RNAi Strategies for Crop Protection—A Review

**DOI:** 10.3390/molecules28062700

**Published:** 2023-03-16

**Authors:** Nurzatil Sharleeza Mat Jalaluddin, Maimunah Asem, Jennifer Ann Harikrishna, Abdullah Al Hadi Ahmad Fuaad

**Affiliations:** 1 Centre for Research in Biotechnology for Agriculture (CEBAR), Universiti Malaya, Kuala Lumpur 50603, Malaysia; 2 Peptide Laboratory, Drug Design & Development Research Group (DDDRG), Department of Chemistry, Faculty of Science, Universiti Malaya, Kuala Lumpur 50603, Malaysia; 3 Institute of Biological Sciences, Faculty of Science, Universiti Malaya, Kuala Lumpur 50603, Malaysia

**Keywords:** RNA interference, non-transformative, exogenously applied dsRNA, biopesticide, delivery system, nanocarrier

## Abstract

To fulfil the growing needs of the global population, sustainability in food production must be ensured. Insect pests and pathogens are primarily responsible for one-third of food losses and harmful synthetic pesticides have been applied to protect crops from these pests and other pathogens such as viruses and fungi. An alternative pathogen control mechanism that is more “friendly” to the environment can be developed by externally applying double-stranded RNAs (dsRNAs) to suppress gene expression. However, the use of dsRNA sprays in open fields is complicated with respect to variable efficiencies in the dsRNA delivery, and the stability of the dsRNA on and in the plants, and because the mechanisms of gene silencing may differ between plants and between different pathogen targets. Thus, nanocarrier delivery systems have been especially used with the goal of improving the efficacy of dsRNAs. Here, we highlight recent developments in nanoparticle-mediated nanocarriers to deliver dsRNA, including layered double hydroxide, carbon dots, carbon nanotubes, gold nanoparticles, chitosan nanoparticles, silica nanoparticles, liposomes, and cell-penetrating peptides, by review of the literature and patent landscape. The effects of nanoparticle size and surface modification on the dsRNA uptake efficiency in plants are also discussed. Finally, we emphasize the overall limitation of dsRNA sprays, the risks associated, and the potential safety concerns for spraying dsRNAs on crops.

## 1. Introduction

As the world population is expected to reach 9.8 billion by 2050 [1], global agricultural production has to be increased to meet food needs. Crop pests, pathogens and the diseases they carry are a key challenge for modern agriculture with respect to coping with the rising demand, contributing to up to 40% productivity losses worldwide [2]. Hence, the agriculture industry relies on chemicals (bactericides, fungicides, nematicides and others) to protect crops. According to the Food and Agriculture Organization Corporate Statistical Database (FAOSTAT), the world recorded a high average pesticide use, alone, per unit of cropland between 2010 and 2019. Approximately 2.60 kg of pesticides were applied per hectare over the ten-year period, which equates to a total of 4.9 million tons of pesticides used annually [3]. Although chemical use brings the primary benefit of higher crop yields, pesticide sprays open up Pandora’s box of deleterious environmental and human health problems. For example, chemical pesticides can pollute soil and water, leading to a decline in water quality, harming terrestrial and aquatic species, and negatively impacting soil health [4]. For humans, acute pesticide poisonings accounted for 385 million cases and 11,000 fatalities per year globally [5]. Due to the adverse effects of pesticides, the global agriculture industry has moved towards the aim of reducing crop losses while lowering chemical pesticide use. Several countries, especially the US and Brazil, have grown genetically modified (GM) plants that constitutively produce pesticidal and pathogenic proteins and, arguably, minimized adverse environmental effects [6,7]. However, the development of GM crops typically necessitates 10 or more years of R&D and several million dollars’ worth of investment [8]. More worrying, extreme weather changes have led to the rising expansion of pests and pathogens’ geographic distributions, increased their survival and increased risk of invasion by migratory pests and plant pathogens. Other factors, such as globalization in trading and people’s movement, also pose unforeseen challenges to modern agriculture [9].

Scientists have explored RNAi, which is a specific post-transcriptional cellular mechanism, as an alternative tool for crop protection strategies. RNAi technology allows the manipulation of regulatory mechanisms to silence genes in plant and animal cells using sequence-specific, small double-stranded RNAs (dsRNAs). Mimicking a laser-guided mechanism in precision, dsRNAs are produced by copying complementary gene sequences of pests and pathogens, and these dsRNAs can be used as a template to destroy pests’ and pathogens’ mRNA sequences, ultimately disrupting protein production [10]. Transformative RNAi technology made possible the creation of pest and/or pathogen-resistant transgenic papaya, potato, apple, maize, alfalfa, soybean, tobacco, plum and tobacco [11]. However, similar to the GM approach, the development of transgenic RNAi crops can be challenging due to stringent regulations, lengthy development timelines and intensive resource commitment that is required before commercialization [11].

As an alternative to the transgenic approach, scientists have explored the use of naked dsRNAs, which can be applied directly onto plants without inserting plasmid DNAs. Also referred to as spray-induced gene silencing (SIGS), the first report of exogenous dsRNAs in plants was carried out by mechanically inoculating bacterially produced naked dsRNAs on pepper mild mottle virus (PMMoV) infected plants to induce RNAi effects for the triggering step of post-transcriptional gene silencing, thereby targeting viral RNAs for degradation [12]. Besides using bacterial systems, these naked dsRNAs can be synthesized in vitro using T7-RNA polymerase [13] or via chemical synthesis [14]. However, these naked dsRNAs are relatively short-lived and susceptible to nuclease degradation upon exposure to UV radiation, in-plant environment, soil and water environment [15,16], unless they are encapsulated [17] or layered with nanoparticles [18].

Another important challenge for spray-on applications of dsRNAs is the unique barriers of walled plant cells. The plant cell wall is composed of a tough semipermeable matrix that serves not only as a protective barrier to the external environment but also as a barrier to the uptake of foreign materials into plant cells [19]. While the plant cell wall permeability may be dynamic in nature, previous studies suggested that a plant cell wall size exclusion limit ranges between 5 and 20 nm [20]. The relatively small pore sizes would likely restrict the movement of larger nanoparticles, thus preventing delivery of encapsulated dsRNAs into and throughout the plant. Several barriers to RNAi-mediated control, such as poor cellular uptake, high levels of dsRNAs degradation and absence of systemic RNA transport have been reviewed in detail by Joga and colleagues [21], and more recently by Bennett and colleagues [22].

One possible approach to solving challenges associated with impaired endosomal escape, dsRNA degradation and poor cellular uptake of dsRNA is by using nanocarriers as a delivery vehicle for dsRNAs [23]. Nanocarriers, as defined by the Union of Pure and Applied Chemistry (IUPAC), is a particle of any shape with dimensions in the range of 1–100 nm. In this review, we present recent developments in various nanomaterial mediated biomolecule (nanocarrier) delivery strategies for plant systems. The focus of this review is to discuss the effects of these design variables, in particular nanoparticle sizes and surface modifications, on the dsRNA uptake efficiency and biological effects within plant cells. This review also summarizes the purpose of using each nanocarrier based on three parameters, namely, RNA size, nature of target and method of delivery. Finally, we highlight the limitation of topical RNAi technology, the importance of technological risk and safety evaluation, and potential future research avenues in this domain.

## 2. Non-Transformative RNAi Strategies

Recent publications on non-transformative RNAi strategies were reviewed by searching the Thompson Reuters Web of Science (WoS) database using combinations of search terms (Queries), asterisks and Boolean operators. Queries were as follows: (1) TS = (topical*) AND TS = (*RNA silencing); (2) TS = (topical*) AND TS = (*RNA*) AND TS = (crop); (3) TS = (topical*) AND TS = (*RNA*) AND TS = (plant). This selection was further refined to include research articles only that were published between 2017 and 2022. The initial search found 1631 journal publications. Next, duplicated entries were removed, and abstracts of the papers were reviewed manually to filter publications that met two inclusion criteria: (1) research articles and (2) methodological approach that uses nanoparticles to deliver RNAs into plant cells. The filtering process resulted in 16 relevant publications that experimentally investigated potential applications of topical dsRNA sprays on crops.

**Figure 1:**
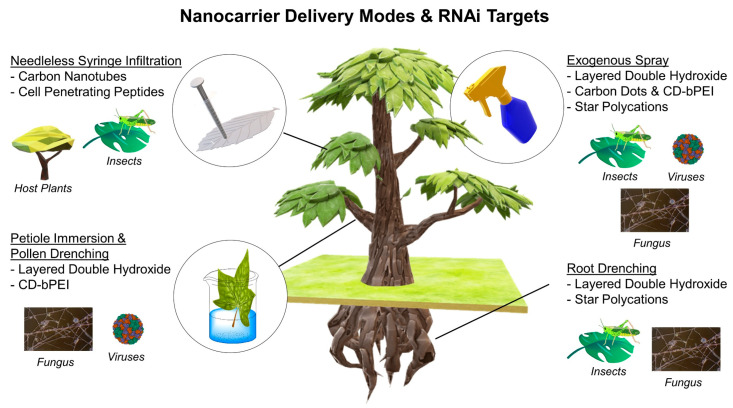
Overview of the recent nanotechnologies for RNAi delivery in plants, its delivery modes and the RNAi targets. The Viruses image is taken from Protein Data Bank (PDBID = 1F15) and the *Fusarium solani* (the Fungus image) was taken by Josef Reischig under CC BY SA 3.0 licensed to Wikimedia Czech Republic. Color image available online.

These publications described seven novel designs of nanocarriers (delivery vehicles) for RNAs into plant cells. Important details, such as nanoparticle design, RNA silencing activity and mode of delivery are presented in Table 1 and Table 2. The summary of the nanotechnologies is also represented in Figure 1 and is further discussed in the following sections. Additionally, other molecules that were not identified using our search queries will not be discussed herein (such as engineered polymer nanoparticles). A collective example of engineered nanoparticles that have been reviewed recently is by Pugsley and colleagues [24].

### 2.1. Layered Double Hydroxide (LDH)

**Figure 2:**
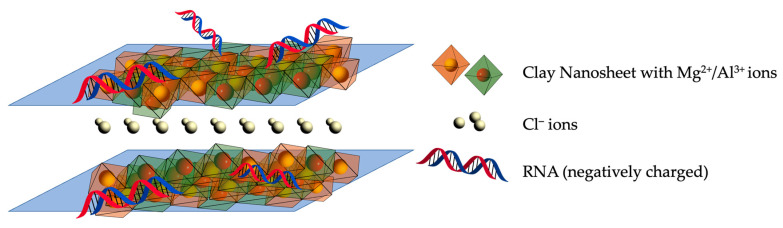
Intercalating LDH nanosheets (magnesium/aluminum ions clay) and chloride ions form a strong layered structure. The metal cations capture dsRNA (R-PO_4_^2−^) via strong ionic interactions. Color image available online.

Layered Double Hydroxides (LDH, Figure 2) are hydrotalcite-like, 2D-ionic lamellar nanoparticles that consist of positively charged layers. A general formula of an LDH is [M^2+^_1−x_M^3+^ _x_(OH)_2_][A^n−^]_x/n_·zH_2_O, where M^2+^ and M^3+^ are divalent and trivalent metal ions, and A^n–^ is the interlayer charge-balancing anion. A great amount of work has demonstrated the multi-purpose nature of LDH including as a biocompatible, low-toxic transporter for gene and drug delivery in mammalian cells [35]. More recently, LDH also demonstrated its capability as a transporter of genetic materials and biologically active compounds into intact plant cells [18,25,26,36].

The uptake of LDH by plant cells has been reported as both by free penetration as well as by clathrin-mediated-endocytosis (CME), in which the LDH nanosheets are engulfed by clathrin-coated vesicles before they are released and dispersed within cytosols [36]. As the translocation of extracellular materials into plant cell walls is limited by the pore diameters, size becomes a key factor for the successful internalization of LDH nanoparticles in plant cells. RNAs encapsulated by LDH nanoparticles were shown to be best suited to deliver long dsRNAs targeting essential genes in viruses and fungi that affected host plants [18,25,26]. In a study carried out by Yong and colleagues [25], LDH nanoparticles with average diameters of 30 and 50 nm displayed the most rapid internalization within pollen cell walls, which in turn influenced gene silencing effects in the target Cucumber Mosaic Virus (CMV). LDH also has the advantage of protecting fragile naked dsRNAs from degradation and thus ensuring prolonged silencing effects post-spray [18].

Methods of delivery for long dsRNAs encapsulated by LDH nanosheets were shown to influence RNAi efficiency against a target. For example, spraying of dsRNAs-LDH resulted in the highest reduction of fungal disease severity compared to leaf petiole adsorption and root dipping [26]. High-pressure spraying and petiole adsorption were effective in controlling Fusarium crown and root rot, as the non-processed dsRNAs are present in xylem vessels and the apoplastic space, thus avoiding plant DICER-LIKE processing. Eventually, the dsRNAs will be consumed intact by the fungi and cleaved by fungal DICER proteins into siRNA, which leads to a better capacity for systemic RNAi against the target fungal genes [37]. The same concept applies when eliciting RNAi effects against insects to allow apoplastic delivery of RNAs. However, unlike fungi and insects, triggering RNAi effects against viruses and endogenous plant genes would require RNAi to occur inside the plant cell and allow symplastic RNA delivery, which can be achieved via high-pressure spraying [37].

Although LDH uptake was reported in several plant parts including leaves [18,26] and pollen grains [25], further studies after dsRNA treatment, such as short RNA- sequence profiling (sRNA-seq) in various plant parts [38], should be carried out to understand uptake and systemic protection mechanisms conferred by LDH in plant cells.

### 2.2. Carbon Dots (CDs)

**Figure 3:**
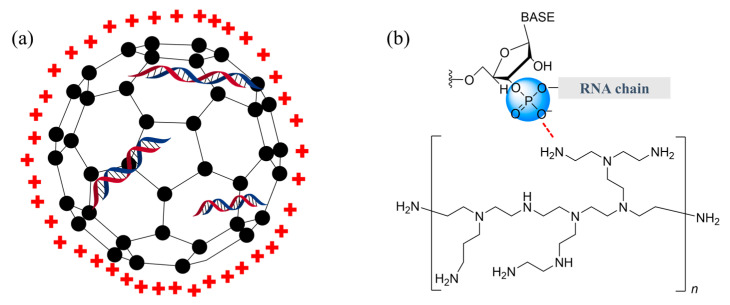
(**a**) Representation of RNAs contained inside carbon dots and (**b**) PEI-RNA interaction. Red-plus sign (**+**) represents positive charges on the surface of carbon dots. Black dots (•) represents carbon atom. Color image available online.

Much interest in carbon dots (CDs, Figure 3a) in plants has evolved around the unique optical properties, which have led to several translocations and uptake studies of CDs in plant cells [39,40,41]. There are reports describing surface modifications of CDs which could impact the uptake and distribution in plant cells. The surface of CDs can be modified, for example, by functionalizing CDs with polyacrylic acid (PAA) and polyethyleneimine (PEI, Figure 3b) to yield positively charged (CD-PEI) and negatively charged (CD-PAA) CDs [39]. Surface modification on CDs changed their size, allowing tailor-made CDs to transverse across the plant cell wall that has a size exclusion limit of between 3 and 10 nm in diameter [42].

CDs are also versatile as a delivery vehicle of genetic materials into plant cells. A recent study reported that amine-functionalized CD (CD-PEI) is suitable as a nanocarrier for siRNA to silence transgenes and endogenous plant genes [27]. Using application methods such as high-pressure spraying or needle-less syringe infiltration, the methods resulted in symplastic RNA delivery that silences transgenes and endogenous plant gene expression [37]. Moreover, CDs were shown to be capable of successfully protecting siRNA from nucleases, with minimal degradation reported after a one-hour incubation with RNase-III [27]. Nuclease protection is attributed to the binding between positively charged amine-functionalized CDs bound and negatively charged polyphosphate groups of nucleic acids [43]. The efficiency of CD-PEI-siRNA in silencing target genes in plant cells depends on several factors, particularly the size of CDs. A limited amount of silencing activity was observed for the largest CD-PEI-siRNA (having an average hydrodynamic diameter of 8.7 nm) while a much higher silencing activity was reported for the intermediate-sized CD-PEI-siRNA (having an average hydrodynamic diameter of close to 3.9 nm). However, the smallest-sized CD-PEI-siRNA (having an average hydrodynamic diameter near 1.1 nm) also displayed limited silencing activity. These observations showed that, in addition to the size exclusion limit of the cell wall, the efficiency of silencing activity depends on other barriers in the cellular system, such as endosomal escape [27,44], endocytosis and release of siRNA [27]. Finding a plausible explanation for the influence of these barriers on silencing efficiency needs further research.

### 2.3. Carbon Nanotubes

**Figure 4:**
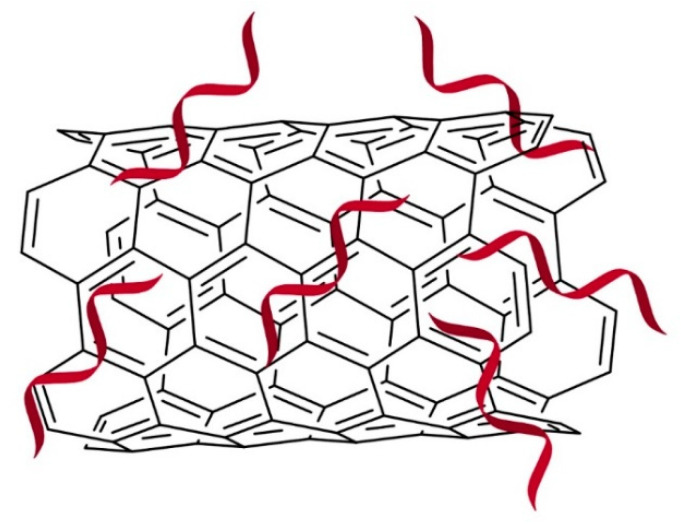
Surface modified CNT (cations) attracts and ‘captures’ negatively charged RNA strands. Color image available online.

Carbon nanotubes (CNTs, Figure 4) are cylindrical hollow nanomolecules that are hydrophobic in nature. Because of the hydrophobic property, unmodified CNTs are less biocompatible, and it is unlikely that they can be integrated into biological systems unless they undergo functionalization or surface modification [45]. The first use of CNT as a plant gene delivery vehicle was reported in a study with Nicotiana tabacum cells in which oxidized-single wall carbon nanotubes (SWNTs) were conjugated with fluorescein isothiocyanate and single-stranded DNA, with the aim to penetrate cell membranes and intact plant cell walls without using a gene gun [46]. Although the study demonstrated the potential of CNT conjugates to deliver DNA and small dye molecules into walled cells, the internalization mechanism of SWNTs in intact plant cells has not been studied in great detail. Besides SWNTs, other studies have explored the capability of multi-walled carbon nanotubes (MWNTs) to penetrate plant cells. For example, a study by Serag and colleagues [47] elucidated the multiwalled carbon nanotube (MWNT) internalization mechanism into plant protoplasts using TEM and confocal imaging techniques. The findings suggested that MWNT uptake by plant protoplasts is facilitated by an endosomal escape mode while their translocation into key cellular structures is size-dependent.

Subsequent studies have elucidated the ability of CNT as a carrier of other biomolecular cargoes into intact plant cells, for example, cellulase [47], plasmid DNAs [29,48,49,50] and siRNAs [29]. These biomolecules bound non-covalently on CNTs based on pi-pi stacking, enabling the biomolecules to be efficiently internalized into walled plant cells. Interestingly for naked RNAs that are easily cleaved by nucleases, their adsorption to CNTs delayed intracellular RNA degradation and prolonged their silencing effects [29]. Similar to CDs, CNTs were shown to be suitable as an siRNA carrier of transgenes and endogenous plant genes that allow RNAi effects to occur inside the plant cells via symplastic RNA delivery. The use of non-charged CNT surface was shown to alleviate cellular toxicity problems that are commonly observed when delivering negatively charged RNAs on positively charged vehicles; however, full desorption effects of CNT were not explored after 3 h post-infiltration [29]. For future studies, a long-term evaluation on toxicity and desorption or decomposition of CNT in plants is desirable to fully demonstrate the safe use of CNT as a carrier for dsRNAs in agriculture.

### 2.4. Chitosan

**Figure 5:**
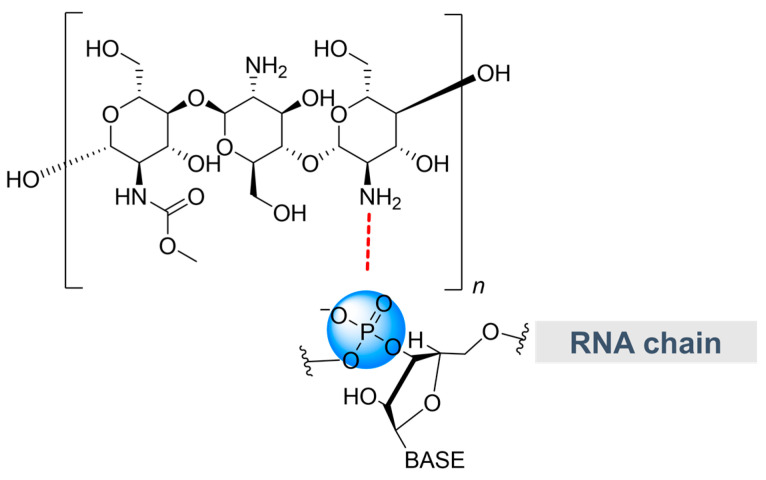
Binding interaction (dotted line in red) between polymeric chitosan and RNA. Color image available online.

Chitosan (Figure 5) is a deacetylated form of biopolymer chitin and is composed of random copolymers (β1→4) 2-amino-2-deoxy-D-glucopyranose (GlcN) and (β1→4)-2- acetamido-2-deoxy-D-glucopyranose (GlcNAc) repeating units [32]. Chitosan is well known for its versatility as it can be easily modified chemically to add desired functionalities. For example, by adding a mild acidic solution to chitosan, the biopolymer would carry positive charges on its amino groups since -NH_2_ is protonated and forms -NH_3_^+^. The positive charges elicit electrostatic interactions with negatively charged phosphate backbones of nucleic acids when mixed in solution, and genetic materials are encapsulated in stable nanostructures (nanoparticles) for efficient gene delivery [51].

While there is a larger body of research on the use of chitosan carriers in animal cells, which have shown good protection against nucleases [52,53,54], a few studies have demonstrated the potential of chitosan nanoparticles as a carrier to transport genetic material into walled plant cells [32,50]. Recent work investigating the physicochemical characterization of the dsRNA-chitosan complex reported a greater binding affinity between the negatively charged phosphate groups from the RNA and positively charged methyl groups of chitosan at a ratio (N/P = 1). The dsRNA-chitosan complex also displayed low toxicity profiles when evaluated against lettuce and human red blood cells, and therefore could be a future candidate for crop protection and improvement strategies [32]. Another study on chitosan-SWNT hybrid nanoparticle demonstrated that the nanoparticle can enhance the loading and trafficking efficiency of the plasmid DNA into plant chloroplasts [50]. The deacylated chitosan was designed to be covalently bonded onto the carboxylated-SWNTs to afford more stable chitosan functionalization in a plant system [50].

### 2.5. Peptides

The topical application of peptides as the nucleic acid carrier has been one of the major focuses for delivery in plants. Peptides are short chains of amino acids that carry positive charges on the cationic groups. The positive charges interact electrostatically with negatively charged nucleic acids to encapsulate the genetic materials for an efficient delivery [17,55,56]. Peptides also possess cell-penetrating properties that enable the translocation of genetic materials across plant cell walls [57]. For example, branched amphiphilic peptide nanoparticles (BAPC) were studied in several formulations (ranging from 25 to 100 nm), to confirm dsRNA uptake by pea aphids through oral feeding [58]. Inclusion of BAPC-dsRNA in the aphid diet was found to suppress BiP and ARMET gene expression in *Tribolium castaneum* and *Acyrthosiphon pisum,* consequently impairing protein folding and resulting in premature death of these plant pests. More importantly, ingestion of BPAC-dsRNA showed a lethality rate of 6 to 9 days earlier compared to feeding with dsRNAs alone, suggesting BPAC complexation enhanced oral delivery of dsRNAs and resulted in improved RNAi effects.

**Figure 6:**
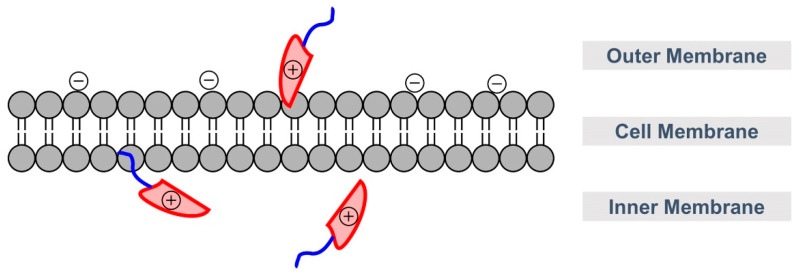
CPP (polycation, in red) passing through cell membrane (negatively charged), carrying its cargo (in blue). Color image available online.

In plant cells, functionalizing cell-penetrating peptides (CPP, Figure 6), for example, Bp100 (having an amino acid sequence: KKLFKKILKYL), with positively charged peptides helped the CPP to effectively condense and translocate genetic materials across cell walls. For example, conjugation of CPPs with poly-lysines (KH)_9_ allows the CPPs to have a greater functional presence at the surface of the genetic material-CPP complexes [17]. The design strategy has facilitated better penetration of dsRNAs across cell walls and plasma membranes.

### 2.6. Gold Nanoparticles

Gold (Au) refers to the solid inorganic aurum metal nanoparticle that has been used in various molecular delivery applications [30,43]. Au nanoparticles have received wide attention for biomedicinal applications primarily due to their biocompatibility. Not only that, the synthetic approach to producing gold nanoparticles allows one to produce nano-sized particles in addition to the ease of surface functionalization or modification [59]. For example, several chemical functional groups, such as COOH, S and NHS, were reported to be able to easily form coordinate bonding, thus allowing for polyionic gold nanoparticle surface modification and thereafter having the potential for complexation with polymeric nucleic acids such as siRNA [30,60]. A similar approach was conducted by Elhaj Baddar and colleagues [61] using an inorganic material, calcium phosphate. To the best of our knowledge, the only example of the use of Au nanoparticles to deliver siRNA was reported by Zhang et al. [31] where the group functionalized Au with polyethyleneimine (PEI) to produce 6 to 27 nm Au-PEI nanoparticles. Zhang and colleagues demonstrated that the constructs were able to deliver 21 bp siRNA and silence the mGFP5 transgene in *Nicotinum benthamiana* while being non-toxic to the host plant.

### 2.7. Other Potential Carriers—Silica and Liposomes

**Figure 7:**
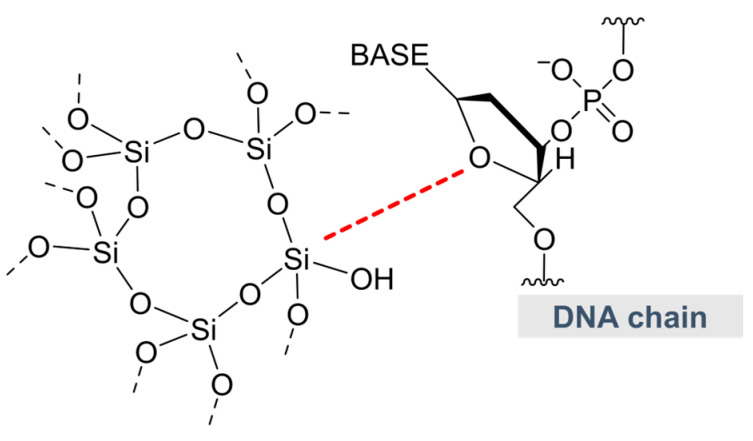
One of many Si-O interactions (dotted line in red) between SNP and DNA strand. Color image available online.

Silica nanoparticles (SNP, Figure 7) have promising physicochemical and thermal stability and are known for their high loading capacity due to porosity [62]. Uptake of SNP by mammalian cells is well documented in biomedical fields with the nanoparticle uptake efficiency being found to be dependent on surface charges [63] and particle sizes [64]. In addition, several works have reported the capability of mesoporous silica nanoparticles to deliver DNA into intact plant cells as a tool for transient gene expression [65,66,67,68] or to transport phytochemicals into plants [69]. However, to our knowledge, no work has been carried out to explore the use of silica to deliver RNAs in plant cells.

**Figure 8:**
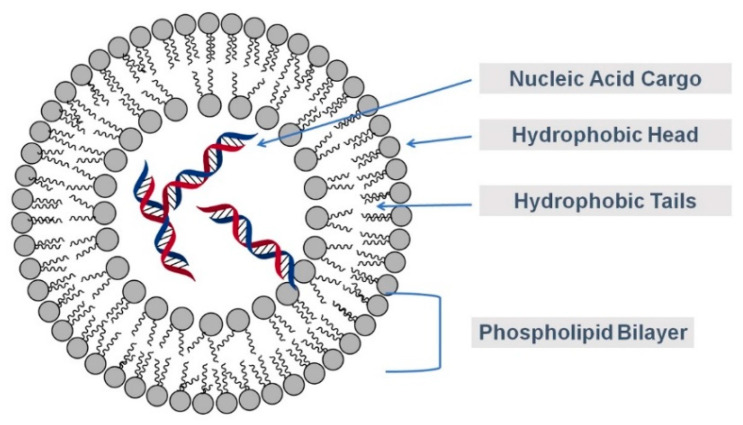
Representation of captured DNA in a liposome. Color image available online.

Liposomes (Figure 8) are surfactants that are made of various types of phospholipids and can serve as a vesicle to penetrate plant cell walls. Previously, liposomes have been demonstrated to be effective at delivering nutrients using a 100 nm PEGylated liposome into tomato leaves [70]. The plant cell body was stained with fluorescein, which is the loaded dye used to visualize the foliar uptake of the nanoparticles using confocal microscopy [70]. Several works have explored the use of liposomes for transgenic expression of dsRNAs in plants and as an artificial diet or for feeding experiments in insects, fungi, bacteria and viruses [71]. Liposomes and exosome-like liposomes were also utilized as nanoparticle carriers in CRISPR/CaS [72], but to the best of our knowledge, there is no published report on the use of liposomes for transporting RNAi cargos into plant cells.

## 3. Potential Risks, Safety Concerns and Limitations

An important aspect of topical RNAi application is the need for risk assessments and management of the dsRNA-based products. Several key aspects for consideration for risk assessments have been described in reports such as the US EPA’s “White paper on RNAi technology as a pesticide: Problem formulation for human health and ecological risk assessment” [73] and the Organization for Economic Co-operation and Development working paper “Considerations for the Environmental Risk Assessment of the Application of Sprayed or Externally Applied dsRNA-Based Pesticides” [74]. The risks of topical RNAi are unique, differing from those of conventional genetic modification, since dsRNAs can be applied as an active ingredient in biopesticides, thus presenting risks similar to traditional pesticides. Skin and respiratory irritation or damage and potential environmental contamination are some risk assessment and management aspects that need to be considered if these nanotechnologies are to be commercialized. However, the particular concerns relating to RNAi silencing activities are potential off-target silencing effects on target and non-target organisms that could be elicited with sufficient sequence similarity between dsRNA and off-target transcripts [75,76]. Addressing the concern of possible off-target effects is especially important to ensure public buy-in as the technology reaches the market. Therefore, designs of RNAi target sequences should be highly specific and have no homology and negligible sequence similarity with off-target transcripts, to minimize off-target hits.

Bioinformatics tools and models have been particularly useful in designing RNAi targets and predicting potential off-target predictions. Reliable searching, predictions and designs of RNAi triggering sequences were made possible with the availability of genomic data libraries of numerous species such as the Drosophila RNAi Screening Center (available at http://www.flyrnai.org; accessed on 29 November 2022) [77] and Genome RNAi (http://www.genomernai.org; accessed on 29 November 2022) [78], web-based design tools including dsCheck (http://dscheck.rnai.jp; accessed on 29 November 2022) [79] and OfftargetFinder (https://www.specifly.org; accessed on 21 December 2022) [80], as well as algorithm models such as siRNA-Finder (https://github.com/snowformatics/siFi21; accessed on 29 December 2022) [81], pssRNAit SVM (https://www.zhaolab.org/pssRNAit; accessed on 26 December 2022) [82] and PFRED (https://github.com/pfred; accessed on 21 December 2022) [83]. Nonetheless, bioinformatics prediction should be supplemented with empirical data from feeding assays on selected test species taxa to verify off-target effects and support risk assessments [84]. The selection of test species may be based on three indicators, which are the sensitivity of the taxa, the representativeness of the test species for a valued taxon, and the availability and reliability of the test species and test protocols with respect to detecting adverse effects on the relevant risk assessment criteria [76].

There also remain uncertainties over the possible fates of dsRNAs once they are translocated into the target cells. For example, considering clathrin-dependent endocytosis is a highly conserved mechanism across eukaryotic species [85], there is a possibility that dsRNA can be internalized into the cells and potentially lead to innate immune response activation by long dsRNAs [71,86]. Furthermore, the risks of RNAi sprays become more complex as dsRNA-nanoparticle formulation prolongs the stability of the dsRNAs in the environment, soil and irrigation systems. The use of nanoparticles, such as chitosan, may also have unforeseen effects, for example, the suppression of myosin expression in *C. elegans* was reported when chitosan was used as either polyplex nanoparticles or alone [54]. Thus, a rigorous safety assessment is needed to evaluate the potential adverse effects of nanoparticles post-spraying.

The commercial potential for RNAi technologies could be hampered by uncertainties in biosafety regulatory pathways for the technology. While many other countries are yet to clarify their regulatory positions for RNAi sprays, several countries have made the first move in reviewing and defining their regulatory processes. In the USA, the risks of RNAi-based biopesticides are evaluated using chemical pesticide templates while Canada, which adopts a product-based biosafety regulation, oversees topically applied RNAi (e.g., via spraying) based on trait novelty. Australia also has taken a favorable standpoint towards regulating non-transgenic RNAi and approved the proposal to exempt topically applied dsRNAs from GMO regulations (refer to schedule 1A techniques that are not gene technology), while New Zealand ruled that dsRNA-treated eukaryotic organisms do not meet the definition of a GMO (APP203395) [75,87]. Such decisions have facilitated approvals for field trials of dsRNA sprays, namely “BioDirect” technology that controls bee-parasitic Varroa mites (submitted by Bayer/Monsanto) [88], and a biocontrol formulation against Colorado potato beetle, *Leptinotarsa decemlineata* (submitted by Syngenta) [89]. Both field trials are being conducted in the USA.

It is also important to note that one of the commercialization barriers to topical RNAi applications are the high expense of large-scale dsRNA production, especially if the synthesis is carried out using in vitro transcription kits. Private startup firms, for example, GreenLight Biosciences (Medford, MA, USA) and RNAgri (previously known as APSE Inc., St Louis, MO, USA), have developed and patented proprietary dsRNA-mass production methods to lower the cost [11]. Alternatively, microbial fermentation technology can also be used to produce dsRNAs at an economic cost [90]. As these technologies can be used to produce cheap dsRNAs, it is anticipated that the commercial interest in the topical RNAi technology is gaining more traction. The positive trend is evident in our patent landscape analysis that demonstrated the evolving commercial interest in topical RNAi application, with a promising trend in patent publications and patent grants, both covering new methods, formulations and usage of topical dsRNAs in crops (Table 3) [11]. With the evolving patent landscape and encouraging results from the field evaluations of RNAi spray, it is anticipated that the development of carriers for RNA delivery will flourish in the years to come.

## 4. Conclusions and Future Direction

The topical application of dsRNA as active molecules presents a highly versatile crop management strategy that does not require plant transformation methods. Since naked dsRNAs are susceptible to degradation upon exposure to the environment, dsRNAs can be encapsulated or layered with biocompatible nanoparticles to prolong the RNA stability and increase silencing efficiency when applied in field-like conditions. However, the design and selection of RNA carriers will depend on several factors such as the RNA size (e.g., siRNAs and long dsRNAs), the target (e.g., fungi, insects, endogenous plant genes and viruses) and the method of delivery (e.g., trunk injection, spraying, petiole adsorption, etc.).

This work reviewed a selection of nanocarrier delivery systems and their use, such as LDH, CD, CNT, gold nanoparticles, chitosan nanoparticles, silica nanoparticles, liposomes and CPP. The use of these nanocarriers allows dsRNAs to be more stable and efficient when applied in open fields. In perspective, the nanocarriers must first be surface-modified with polar components to allow for carrier-RNA polar interactions. Further studies are also needed to understand the possible toxicity effects of dsRNA-nanocarrier complexes and how various barriers in the cellular system influence the uptake, silencing effects and systemic protection mechanisms in plants.

Despite the promising potential of topical RNAi technology, there are limitations, potential risks and safety concerns in relation to the technology that need to be addressed. Potential off-target effects, uncertainties in the fate of dsRNAs and regulation of the technology, as well as the high production cost of the dsRNAs, may limit the commercial potential of the technology. More importantly, good governance of topical RNAi technology demands a greater corporate responsibility that requires continuous dialogues with relevant stakeholders and that devotes more attention towards addressing ethical issues and societal costs of the technology. To ensure the longevity of topical RNAi application, the authors believe that understanding society’s position on the technology with respect to the aspects of willingness-to-pay, technological adoption trade-offs and public trust are key areas that need urgent attention.

## Figures and Tables

**Table 1:** Summary of various design strategies of delivery vehicles for RNAs. ^a^Chemical synthesis method used for the preparation of the respective delivery vehicles.

No	Delivery Vehicle	Synthesis Method ^a^	Surface Modification	Particle Sizes	RNA Size	Ref.
1	Layered Double Hydroxide (LDH)	Co-precipitation	Mg_3_Al–NO_3_-LDH	80 to 300 nm	330 bp and 977 bp dsRNA hairpin	[18]
		Co-precipitation	Mg_3_Al–NO_3_-LDH	50 to 120 nm	300 bp dsRNA	[25]
		Co-precipitation	Mg_3_Al–NO_3_-LDH	30 to 90 nm	30–40 bp dsRNA	[26]
2	Carbon Dot (CD)	Solvothermal	Branched Polyethyleneimine	2.7 to 3.9 nm	22 nt siRNA	[27]
	CD-Branched Polyethylenimine (bPEI)	Hydrothermal	Lipid modification (addition of 1,2-epoxytetradecane)	220 nm	250 bp dsRNA	[28]
3	Carbon Nanotube (CNT)	HiPco	Not reported	776 nm (length), 1.567 nm (height)	19 nt siRNA	[29]
4	Cell-penetrating peptide (CPP) (i.e., Bp100)	Chemical synthesis	Polycation (KH)_9_	100 to 300 nm	456 bp dsRNA	[17]
5	Gold (Au) Nanoparticle	Chemical synthesis	Poly-l-arginine	60 to 100 nm	355 bp dsRNA	[30]
		Chemical synthesis	Polyethyleneimine	6 to 30 nm	21 bp siRNA	[31]
6	Chitosan Nanoparticle	Chemical synthesis	Hydrochloric acid (HCl)	73.25 nm	40 bp DNA producing 21 nt ssRNA	[32]
7	Star Polycation (SPc)	Chemical synthesis	hpRNA-SPc	Not reported	331, 333, 413 and 508 bp hairpin dsRNA	[33]
			dsRNA-SPc	Not reported	359 and 489 bp dsRNA	[34]

**Table 2:** Summary of mode of delivery for RNAs, its targets or desired effects.

No	Delivery Vehicle	Mode of Delivery	Target/Effect	Exposure	Durability	Efficacy	Ref.
1	Layered Double Hydroxide (LDH)	Topical application on *A. thaliana* leaves or spray atomizer on *V. unguiculata* and *N. tabacum*	Viruses. Silences replicase gene of PMMoV and target gene of CMV	200 μL samples of 15 μg CMV2b-dsRNA–LDH, sprayed at day 0 only	Partial degradation was observed for naked dsRNAs after 2 min while dsRNA-LDHs remain intact	LDH-only treated plants developed more necrotic lesions compared to dsRNA-LDH at the same time points (Day 1 and 5). LDH-dsRNA offered higher protection against the virus at 20 days post spraying	[18]
		*S. lycopersicum* pollen drenching	Virus. Silences target gene of CMV	Concentrations of LDH-50 and dsRNA were 100 and 10 mg/L. Treatment is up to 7 days	Complete degradation for naked dsRNAs after 10 min while dsRNA-LDHs remain intact	Treatment for 3 days with LDH–dsRNA led to a 16.7% decrease in GUS protein activity. No significant changes were observed with naked dsRNAs alone after treatment for 7 days	[25]
		Leave spray, petiole adsorption or root dripping	Fungus. Silences FoCYP51, FoChs1 and FoEF2 genes of *Fusarium oxysporum*	Leaves spray & petioles adsorption: 300 μg of dsRNAs in 3 mL of ddH_2_O per plant Root dipping: 3 μg of dsRNA in 3 mL of nano solution per plant	Degradation of naked dsRNA began after 1 min and completed after 10 min. dsRNA bounded LDH is still intact after 1 h of incubation	Disease severity that was observed for leaves spray (10%), petioles adsorption (15%) and dipping roots (35%)	[26]
2	Carbon Dots (CD)	Low-pressure spray	Host Plant. Silencing *GFP* transgenes and endogenous genes in *N. benthamiana* and *S. ycopersicum*	Concentration of siRNA/CD is 12 ng/μL, and is sprayed on plants at Days 1, 7 and 14	Complete degradation of naked dsRNAs in 15 min. dsRNA-CDs remain intact after a 60-minincubation	A 79% reduction was observed in the phenotypic tissues at Day 5 after treatment. Bleaching phenotype persisted up to 20 days after treatment	[27]
3	CD-Branched Polyethylenimine (CD- bPEI)	Leave spray and petiole immersion	Virus. Silences RNA polymerase and coat protein genes of Grapevine leafroll associated virus-3 (GLRaV-3)	A 0.00092 g/mL and translatedinto a 32x dilution factor	Degradation of naked dsRNAs began after 2 h while dsRNA-CDs-bPEI remain intact	Virus titre decreased over three weeks after a single-dose administration, but multiple doses are needed to improve fruit quality	[28]
4	Carbon Nanotube (CNT)	Needleless syringe infiltration on leaves of *N. benthamiana*	Host Plant. Silences mGFP5 transgenes in leaves	Concentrations: siRNA (100 nM) SWNT (2 mg/liter)	Degradation (94%) of naked dsRNAs after 6 h. dsRNA-SNWT degradation (30%) after 6 h	Gene silencing efficiency was up to 95% within 1 day after infiltration	[29]
5	Cell-penetrating peptides (CPP) (i.e., Bp100)	Needleless syringe infiltration on *A. thaliana* leaves	Insect. Silence GFP and firefly luciferase genes	100 μL of the dsRNA-peptide, incubated for up to 36 h	Naked dsRNAs were slightly degraded after 12 h while the dsRNA-peptides remain intact	No silencing effects was observed for naked dsRNAs while genetic down-regulation was observed for dsRNA-peptides within 12 h and up to 36 h	[17]
6	Gold Nanoparticle	Not tested on plants (insect cell assay only)	Insect. Silences Luciferase gene in *Spodopteria frugiperda*	dsRNA (50 μg/mL)	dsRNA-Au showed better endosomal escape compared to dsRNA alone.	Up to 58% reduction of the luciferase activity for dsRNA-Au compared to dsRNA alone	[30]
		Needleless syringe infiltration on mGFP5 *N. benthamiana* leaves without needle	Host Plant. Silences mGFP5 transgenes in *N. benthamiana* leaves	100 ng of siRNA	Complete degradation was observed after 30 min of incubation for naked dsRNAs while dsRNA-Gold NP remain intact	No upregulation of NbrbohB suggests low to no stress to plant tissues	[31]
7	Chitosan Nanoparticle	Not tested	Virus. Silences coat protein gene of *Tomato mosaicvirus*	200 μg/mL of the dsRNA-chitosan.	Not reported	dsRNA-chitosan has low toxicity with no inhibitory effects on root development	[32]
8	Star Polycation (SPc)	Spray on oilseed rapes leaves infested with *Myzus persicae* using pneumatic water sprayer	Insect. Silences essential genes. ATP-A: 413 bp, LOC111039523; ATP-d: 383 bp, LOC111041166; ATP-G: 301 bp, LOC111040044 of *M. persicae*	0.2 μL dsRNA/SPc formulation sprayed at Day 0	Complete degradation was observed for naked dsRNAs in 12.5% of aphid hemolymph after 1.5 h while dsRNA-SPc remain intact.	Control efficacy was 61% on Day 3 after treatment with SPc-dsRNA and maintained at 50% until Day 6.	[33]
		Root drenching	Insect. Silencing *M. persicae* vestigial (vg) & Ultrabithorax (Ubx) genes involved in wing formation.	Exposing radish seedling to 200 μL dsRNA/SPc formulation at Day 0 prior to *M. persica* transplantation	Not reported	About 40% of *M. persica* developed effective wings when both dsRNA-genes were used	[34]

**Table 3:** Patent Families on Methods of Delivery and Compositions to Introduce Exogenous dsRNAs into Plant Cells.

Application Number	Priority Date	Legal Status	Assignee	Invention Details
*US15/579,120*	03.06.2015	Granted in US (2020)	Monsanto Technology LLC	*Composition*: Polynucleotide, particulate and osmolyte *Delivery*: Abrading a surface of a plant with a particulate, followed by applying an RNA onto the plant surface
*US15/579,125*	02.06.2015	Granted in US (2021), EP (2021)	Monsanto Technology LLC	*Composition*: Polynucleotide, at least one lipase enzyme, one or more osmolytes, surfactants, abrasives or any combination *Delivery*: Applying lipase enzyme, osmolytes, and surfactants, followed by an RNA onto the plant surface
*US16/062,008*	14.12.2015	Granted in US (2021) EP (2021)	Monsanto Technology LLC	*Composition*: Polynucleotide targeting gene of flea beetle and cross-linked cationic polysaccharide *Delivery*: Applying onto a seed, plant surface or foliar spray
*US61/748,095*	01.01.2013	Granted in AU (2019), CN (2019), US (2018)	AB Seeds Ltd./Monsanto Technology LLC	*Delivery*: Soaking ungerminated seed with a solution comprising a concentration of between 0.005 and 1.5 pg/pL of the dsRNA molecule, followed by drying the seed
*US16/583,863*	26.09.2018	Granted in US (2021)	Greenlight Biosciences Inc	*Composition*: dsRNA targeting *Leptinotarsa decemlineata* Inhibitor of Apoptosis (IAP) gene *Delivery*: Spray, fog, seed treatment, drench, drip irrigation, in furrow, insect diet, or bait
*US15/752,274*	13.08.2015	Pending	Forrest Innovations Ltd.	*Composition*: Polynucleotide and at least one cell wall degrading enzyme, a nucleic acid condensing agent, a transfection reagent, a surfactant, and a cuticle penetrating agent
*US14/381,045*	06.03.2014	Granted in JP (2020) US (2020)	RIKEN	*Composition*: Polynucleotide and a carrier peptide containing a cell-penetrating sequence and a penetrating polycationic sequence
*Application* *number*	Priority Date	Legal Status	Assignee	Invention Details
*US15/106,548*	20.12.2013	Granted in AU (2018), CA (2021), EP (2019), ES (2020), US (2020)	University of Queensland	*Composition*: dsRNA and layered double hydroxide with a charge ratio is 2:1 to 1:20 *Delivery*: Spray

## Data Availability

Not applicable.
